# Vascular access challenges in hemodialysis children

**DOI:** 10.1186/s13052-024-01590-4

**Published:** 2024-01-22

**Authors:** Doaa M. Salah, Fatina I. Fadel, Mohamed A. Abdel Mawla, Hesham NAbdel Mooty, Mohamed El Ghobashy, Amr M. Salem, Mohamed Gamal Fathallah, Eman Abobakr Abd Alazem

**Affiliations:** 1 Department of Pediatrics, Pediatric Nephrology and Transplantation Unit, Kasr Alainy Faculty of Medicine, Cairo University, Cairo, Egypt; 2 Department of Pediatrics, National Research Center, Cairo, Egypt; 3 Department of Vascular Surgery, Kasr Alainy Faculty of Medicine, Cairo University, Cairo, Egypt; 4 Department of Radiodiagnosis, Kasr Alainy Faculty of Medicine, Cairo University, Cairo, Egypt

**Keywords:** Challenges, Transhepatic, AVF, Uncuffed/ cuffed CVC, VA survival

## Abstract

**Background:**

Hemodialysis (HD) success is dependent mainly on vascular access (VA). The aim of this study is to share the experience of Pediatric Nephrology Unit (PNU), Cairo University Children’s Hospital (CUCH), with VA-related obstacles in end stage kidney disease (ESKD) HD children.

**Methods:**

This is a retrospective analysis of VA related data of 187 ESKD children received regular HD over 3 year duration (2019–2021). Kaplan–Meier curves were used to present arteriovenous fistula (AVF) and cuffed catheters survivals.

**Results:**

Uncuffed central venous catheter (CVC) was the primary VA for HD in up to 97.3% with 2.7% of patients had AVF performed and attained maturation before initiation of regular HD. Fifty-six (29.9%) patients have inserted 120 tunneled CVCs. AVFs & AV grafts (AVF) were performed in 79 (42.2%) and 6 (3.2%) patients respectively. There were 112 uncuffed CVCs implanted beneath the screen in Rt internal jugular vein (IJV) (44%) Lt IJV (17%), right internal mammary vein (2.7%) while Trans hepatic (TH) technique was used to place 39 uncuffed CVCs (34%) in the inferior vena cava (IVC). Catheter-related bacteremia (CRB) was the most frequent complication in uncuffed and cuffed CVCs (2.58 / 100 catheters day and 10.1 /1000 catheter days respectively). AVFs achieved a high success rate (83%) after 757.71 ± 512.3 functioning days.

**Conclusion:**

Native AVF is the preferred VA for pediatric HD but its creation is limited by the small sized vessels where non-cuffed CVC could be a reasonable relatively long-term alternative. Challenging situations (occluded central veins) could benefit from TH technique of CVC insertion in IVC.

## Introduction

Hemodialysis (HD) was reported to be the most frequently used dialysis modality, in children with end-stage kidney disease (ESKD) [1]. The most difficult aspect of care for pediatric ESKD patients on regular HD is how to obtain an appropriate vascular access (VA) [2].

One of the most challenging tasks is determining which VA option is best for an ESKD child on regular HD. Pediatric age groups, with longer life expectancy, are often exposed to many attempts of VA insertion over their HD period, making the selection of an optimal VA crucial [3].

The permanent arteriovenous fistula (AVF) is the most desirable VA in children on regular HD [4]. Nevertheless, children who use central venous catheters (CVC) as a VA have certain advantages, such as ease of insertion, elimination of the need to wait for maturity as with AVFs, and the avoidance of needle discomfort [5, 6]. When compared to those who have HD through AVFs, children who have HD by CVC have a higher number of complications, more frequent hospitalization owing to infection, and higher morbidity [7, 8].

Because juvenile HD patients have tiny arteries and are subjected to frequent operations and long-term catheterizations, VA problems and failure are too common. Failure of dialysis by conventional VA is a significant medical hurdle that must be addressed by seeking alternate approaches such as trans-hepatic catheters, which is a relatively recent method for inferior vena cava (IVC) and right atrium catheterization [9].

In this study; the authors aimed to report their experience of handling VA related complications and challenges in ESKD children on regular HD at Pediatric Nephrology Unit (PNU), Cairo University Children Hospital (CUCH).

## Patient and methods

**Fig. 1:**
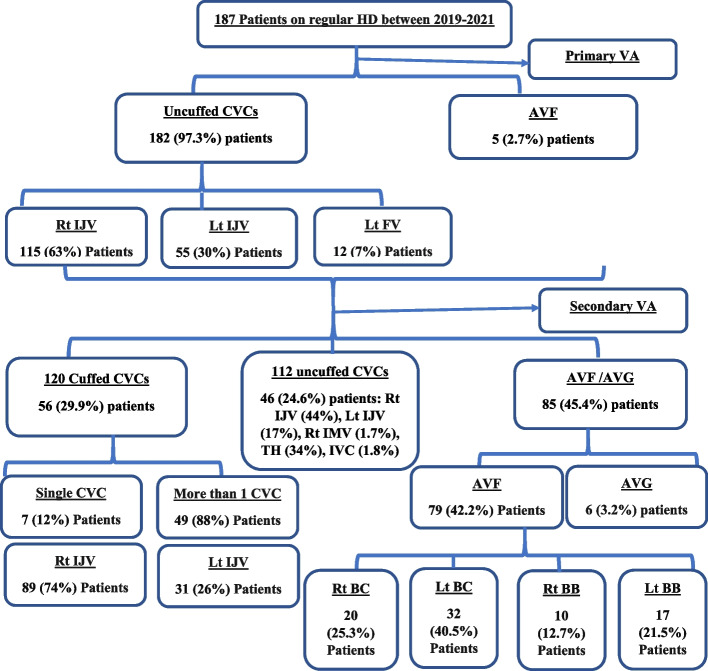
Flow chart of vascular accesses inserted to included patients between 2019–2021

This is a retrospective descriptive study that included eligible ESKD children (actively following up by the end of data collection period) underwent regular HD at HD section of PNU, CUCH over three years between January 2019 and December 2021 (Fig. 1).

A total of 187 patients were enrolled in the study out of 213 children dialyzed at the unit during the study period. Included patients were ESKD children aged between 4.5–12 years underwent regular HD for at least six months with available medical data in the unit.

Being highly specialized tertiary referral center that is designed to handle the challenging relatively low weight children undergoing regular HD; patients are usually referred to other pediatric hemodialysis centers nationwide according to their geographical distribution once they reach 14 years age and 40 kg weight. The study statistics included patients actively underwent VA insertion during data collection duration in addition to patients with cuffed CVCs/ AVF was already inserted/created before the start of the data collection (2019) and then discontinued dialysis by them during the study duration (*n* = 53). Patients referred to other pediatric HD centers, died before the end of 3 year duration of data collection or dialyzed for less than 6 months (total of 26 child) were excluded from the study.

Patients received bicarbonate base dialysis using a high-flux dialyzer, Fresenius 5008 and 4008 classic machines with an average blood flow of 100–140 ml/min, dialysate flow rate of 500 mL/min three times/week targeting 4-h duration for each dialysis session. Patients needed VA for acute indications (acute kidney injury, plasma exchange or other therapies) received their therapies in a separate section other than Hemodialysis section of Pediatric Nephrology Unit called acute dialysis section with separate records and were easily excluded from the study.

The study protocol was approved by Research Ethical Committee, Faculty of Medicine, Cairo University (N-112–2022). Informed consent was obtained from patient’s guardians with all procedures performed according to the Declaration of Helsinki.

Basic demographic data of included patients were obtained. Data of ESKD etiology, dialysis duration, dry weight at initiation of regular HD, and hepatitis C virus (HCV) status were collected. Laboratory data (in the form of serum calcium, phosphorus and hemoglobin level) at the end of study duration were reviewed.

According to Center regulations; uncuffed CVCs are usually implanted, Ultrasound guided, by nephrologist in charge for urgent HD (except in challenging situations, where more senior staff is consulted or patient is referred to Radiologist and meanwhile PD is performed as the only available option of urgent dialysis). Uncuffed CVCs were double lumen 7, 8, 9 and 12 F X 10 and 15 cm manufactured by Amicath Company USA. Cuffed tunneled long-term catheters are usually inserted by the interventional radiologist and sometimes with pediatric vascular surgeon after coordination with pediatric nephrology team. AVFs for patients with appropriate weight/ vessel sized are arranged to be electively created by pediatric vascular surgery. Preliminary preparation for patients planned to insert/ create their VA in the operation theatre (cuffed tunneled CVCs or AVFs) is usually carried out by pediatric nephrology team in PUN. This usually includes optimization of clinical and laboratory data of the patients before undergoing their VA inserted/ created.

Cuffed CVCs were used in young children (less than 9 kg weight) with small veins that couldn’t be punctured by fistula needles and individuals who had failed AVFs and had vascular issues (previous failed/ complicated AVF or originally thrombosed). All tunneled catheters were 8F X 18 cm, 8F X 24 cm or 10F X 24 cm, manufactured by Medcomp Company USA.

Data related to VA was gathered, including type of VA (CVC or AVF), whether cuffed or non-cuffed catheters, the average duration of VA functioning, and the reason for dialysis stoppage using it. Additionally; VA at the time of initiation of dialysis, current and previous accesses and VA related complication were revised. The fate of VA at the end of the study either functioning VA (duration), failed VA or failed with VA related complications were reported.

### Statistical analysis

SPSS software version 19.0 was used for statistical analysis (SPSS, Chicago, IL, USA). Nominal data were expressed as frequencies and percentage, parametric data as means and standard deviations. Non-normally distributed data were expressed as median and interquartile range (IQR). VA characteristics, were measured using descriptive statistics. *P* value less than 0.05 was considered significant.

## Results

The study included 187 patients who actively received regular HD between 2019–2021. Demographic data, primary renal diseases, laboratory and dialysis related data are illustrated by Table 1.

**Table 1:** Demographic data, original renal disease, laboratory and dialysis related data of the study group (*n* = 187)

Variable		
**Sex:**		
Male	N (%)	104 (55.6%)
Female	N (%)	83 (44.4%)
Age (years)	mean ± SD	8.3 ± 3.675
Dry weight (Kg)	mean ± SD	16.6 ± 7.6
**Original kidney disease:**		
FSGS	N (%)	53 (28.5%)
NPHP	N (%)	42 (22.4%)
Obstructive Uropathy	N (%)	41 (21.9%)
PH1	N (%)	16 (8.5%)
Cystinosis	N (%)	9 (4.8%)
aHUS	N (%)	8 (4.27%)
Joubert Syndrome	N (%)	6 (3.2%)
^a^Others	N (%)	12 (6.4%)
**Dialysis duration (months)**	mean ± SD	17.73 ± 10.607
**Laboratory data:**		
Hemoglobin (g/dl)	mean ± SD	10.09 ± 2.789
Serum calcium	mean ± SD	9.78 ± 2.012
Serum Phosphorus	mean ± SD	8.14 ± 3.941
**Comorbidities:**		
Anemia	N (%)	56 (29.9%)
Hypertension	N (%)	63(33.6%)
Cardiomyopathy	N (%)	47 (25.1%)
**HCV status:**		
HCV PCR + ve	N (%)	32 (17.1%)
HCV PCR –ve	N (%)	155 (82.9%)

*aHUS* atypical hemolytic uremic syndrome, *HCV* Hepatitis c virus, *FSGS* Focal segmental glomerulosclerosis, *NPHP* Nephronophthisis, *PCR* Polymerase chain reaction, *PH1* Primary hyperoxaluria type1

^a^Others include: autosomal recessive polycystic kidney disease, Membranoproliferative glomerulonephritis, lupus nephritis and bilateral atrophic kidney

### Uncuffed CVCs

Owing to late referral of the vast majority of included patients PNU for initiation of HD on urgent rather than elective circumstances; 182 (97.3%) patients inserted uncuffed CVCs as their first VA. Only five (2.7%) patients had AVF performed and attained maturation before initiation of regular HD.

Uncuffed CVCs were inserted most frequently in Right internal jugular vein (Rt IJV) (*n* = 115/182) (63%) followed by left internal jugular vein (Lt IJV) (*n* = 55/182) (30%), and left femoral vein (Lt FV) (*n* = 12/182) (7%) (Fig. 1). Uncuffed CVCs served as VA for a mean duration of 19 ± 7.45 days till permanent VA is available.

**Fig. 2:**
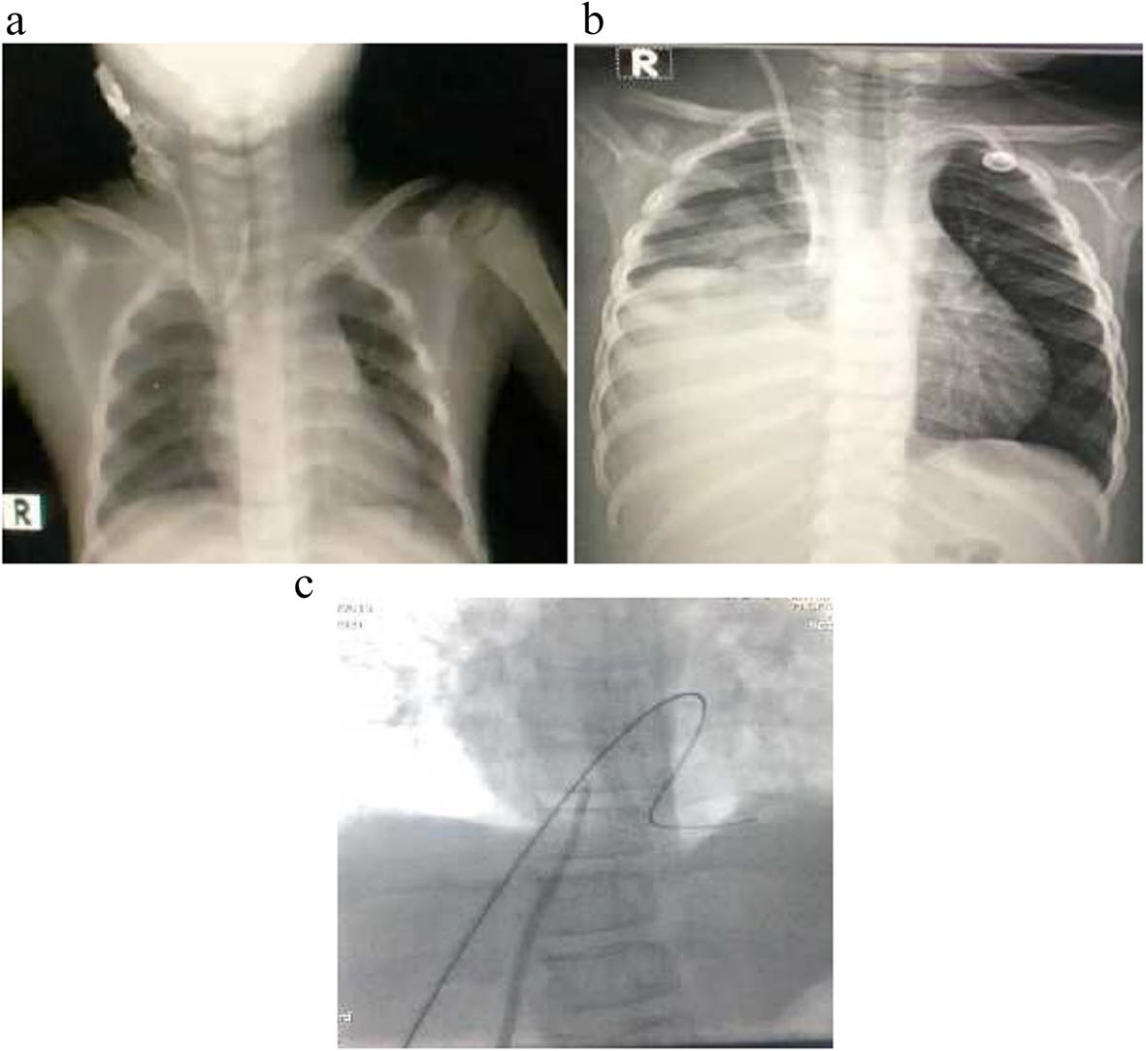
**a** Malposition of catheter in one of the patients. **b** Hemothorax developed in one of the patients after Catheter insertion. **c** Transhepatic catheter insertion in one of the study groups

There were 112 uncuffed CVCs implanted beneath the screen throughout the research period in 46 patients due to the presence of vascular problem as attenuated or thrombosed veins. The most frequent catheters implanted were Rt IJV (*n* = 50) (44%) followed by Lt IJV (*n* = 19) (17%). The Trans hepatic (TH) technique was used to place 39 uncuffed CVCs (34%) in the inferior vena cava (IVC) (Fig. 2c).

In two patients (1.7%), catheters were inserted into the right internal mammary vein (Rt IMV).

Two uncuffed CVCs (1.8%) were placed into the IVC via the left femoral vein (Lt FV), both were terminal patients with thrombosed IJV and hepatic veins.

#### Insertion complications

Insertion complications of uncuffed CVC were reported in 10/ 182 (5.4%) patients. The primary issue we encountered was malposition, in which the catheters went through Rt IJV and subsequently mal-rotated to the innominate vein (*n* = 2/10), subclavian (*n* = 4/10), or other central veins (*n* = 1/10) (Fig. 2a). These catheters were promptly withdrawn. Pneumothorax (*n* = 2/10) and hemothorax (*n* = 1/10) were among the other consequences (Fig. 2b). Before insertion, Venous Doppler study was performed for all of patients.

#### Delayed complications and outcome

As shown in Table 2, the most prevalent consequence with uncuffed TH CVCs was catheter-related bacteremia (CRB) (2.58 per 100 catheters day). Nevertheless; 5 (13%) patients kept their catheters until their date of KT.while the most prevalent complications in uncuffed internal jagular catheters (*n* = 69) were thrombosis 18% (*n* = 13/69) and CRB 17% (*n* = 17/69).

**Table 2:** Complications and outcome of uncuffed and cuffed CVCs among the study group

**Complications and outcome**	**Cuffed tunneled** **CVCs** (*N* = 120) Frequency (%)	**Uncuffed IJ** **CVCs** (*N* = 69) Frequency (%)	**Uncuffed TH** **CVCs** (*N* = 39) Frequency (%)	***P*-value**
CRB	60 (50%)	12 (17%)	15 (38%)	**0.000052**
Perforation	16 (14%)	3 (4.3%)	8 (11%)	**0.033772**
Thrombosis	8 (5%)	13 (18%)	5 (13%)	**0.031264**
AVF maturation	20 (17%)	0 (0%)	0 (0%)	**0.000767**
Transplantation	16 (14%)	9 (13%)	5 (13%)	0.996054

*AVF* Arterio- venous fistula, *CRB* Catheter related bacteremia, *CVC* Central venous catheters

The two patients who inserted internal mammary catheters were died from sepsis.

Regarding patients who inserted IVC catheters after 9 months of dialysis (through which CVC was replaced monthly on a guidewire), one of these patients was transplanted using this catheter, and the other died after 2 years from insertion due to sepsis.

### Cuffed CVCs

Fifty-six (29.9%) patients have inserted 120 tunneled CVCs. Tunneled CVCs were inserted in Rt IJV (*n* = 89/120; 74%), Lt IJV (*n* = 31/120; 26%). Seven out of the fifty-six patients (12%) inserted tunneled CVC once while 49 patients (88%) had their tunneled CVC replaced and swapped over guidewire within the same site of insertion. The mean duration of tunneled CVC usage in HD was 215.37 ± 122.7 days, with a range of 30—310 days.

The most frequent complication encountered with cuffed CVCs was CRB (50%) that was also the leading cause of cuffed CVC removal. Catheter perforation and thrombosis were less frequent complications of cuffed CVCs (14% and 5% respectively). However, 31% of cuffed CVCs were removed due to termination of the indication of their use (AVF maturation or KT) (Table 2). CRB of cuffed CVCs occurred at frequency of 10.1 per 1,000 catheter days. Methicillin-resistant Staphylococcus aureus (MRSA) was the most frequent bacterium infecting cuffed CVCs (up to 60%), followed by methicillin sensitive Staph aureus (28%), and Gram-negative bacteria (6%).

**Fig. 3:**
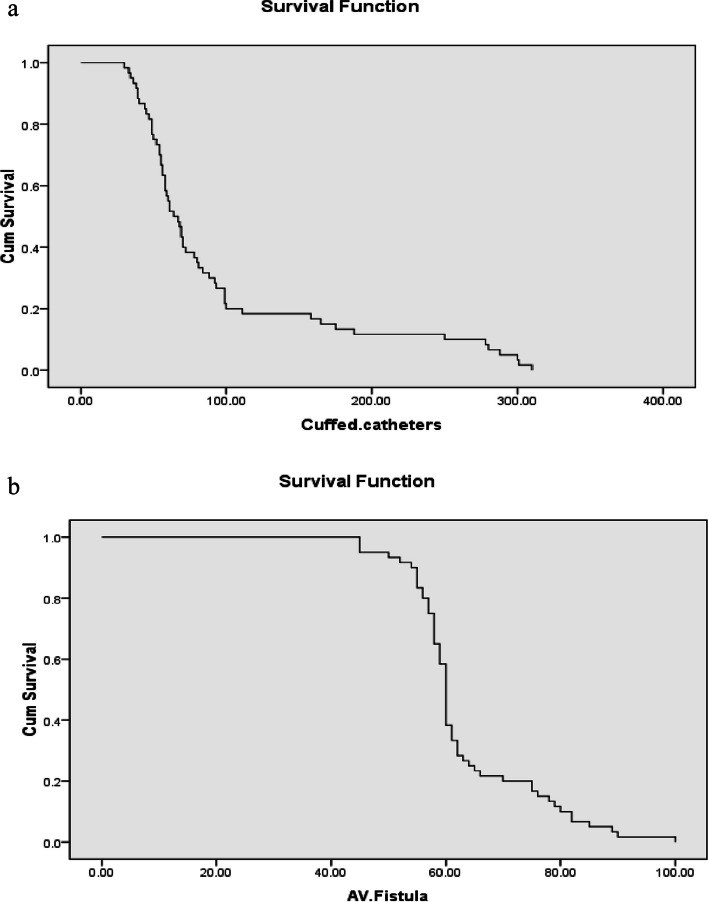
**a** Kaplan Mayer Curve for cuffed central venous catheters survival. **b** Kaplan Mayer Curve for arteriovenous fistula survival

The catheter survival rate and the rate of removal of cases with positive cultures were 19% after 30 days, 15% after 42 days, 49% after 90 days, and 7% after 180 days, respectively. Figure 3 (a) demonstrates Kaplan Mayer Curve for cuffed CVCs survival.

### AVF/AVG

AVF**s** were performed in 79 (42.2%) patients. Left brachiocephalic (BC) was the most common site for AVF creation (40.5%) (Fig. 1). Arterio-venous grafts (AVG) were performed in 6 (3.2%) patients only. As all AVGs were performed using synthetic material, the significant infection incidence associated with them prohibited their frequent utilization as VA in our center.

The mean duration of functioning AVF among the study group was 757.71 ± 512.3 days. Mean time interval between AVF operation and first puncture use (time taken for maturation of AVF) was 61.547 ± 10.082 days.

As indicated in Table 3; AVFs usage was associated with a higher success rate (83%). Nevertheless, several challenges emerged during their use for HD, such as thrombosis in 10 patients; (3 patients had thrombectomy where AVF re-operated again and 7 patients had massive mural thrombosis, making thrombectomy useless). In one instance, bleeding occurred during surgical exploration, but the AVF was spared. Figure 3 (b) demonstrates Kaplan Mayer Curve for AVF survival.

**Table 3:** Outcomes of AVFs (*n* = 79)

	Frequency	Percentage
Functioning AVF	49	63%
Patient transplanted	16	20%
Thrombosis	10	13%
Primary failure	3	3%
Bleeding	1	1%

AVG were failed in 4/6 (66.7%) of patients due to thrombosis in 2 (33.3%) patients and infection accompanied by bleeding in the other 2 (33.3%) patients. Two (33.3%) patients have functioning AVG for 6 and 7 years till the end of data collection.

## Discussion

Kidney transplantation (KT) is the preferred option in children with ESKD. Dialysis is mostly accomplished as a temporary measure until the child is ready for KT. Success of HD is largely dependent on efficiency of VA. Between January 2019 and December 2021, we analyzed VA data of 187 pediatric patients receiving regular HD at HD section of PNU, CUCH.

Several studies have been conducted to analyze VA in pediatric HD population [1–7].

The mean age of our study group was 8.3 ± 3.675 years with dialysis duration of 17.73 ± 10.61 months and weight of 16.6 ± 7.6 kg. Teixiera et al., recently conducted a retrospective analysis of VA for HD with the mean age and weight of their study group were 12.3 years and 38.3 kg respectively [3]. The younger age and smaller weight of our patients make VA more challenging among our study group.

Similar to most of studies of VA and in agreement with the NFK/KDOQI recommendations [10]; Rt IJV was the most frequent site for insertion of uncuffed catheters. Right femoral vein (Rt FV) has been always avoided in trails of CVCs insertions according to center policy. We aimed to keep Rt FV patent being drained to Right iliac vein (Rt IV) that is usually needed for vascular anastomosis during KT. Ultrasound is utilized routinely at our center while placing catheters, and this has given us outstanding outcomes in terms of insertion issues, which were observed in 5% only of our study group. Our findings matched those of previous research [11, 12], which found that success rates of uncuffed catheter insertion ranged from 82- 96%, and recommended that all pediatric clinics should employ ultrasonography for these operations. Malposition was the most prevalent consequence of uncuffed CVCs insertion in the present study, this is similar to previous research on 195 uncuffed CVCs that was done at our facility between March 2002 and June 2005, when malposition was the most common insertion issue [13].

In the present analysis; we employed percutaneous transhepatic (TH) technique to place IVC catheters in 39 patients with insertion success of 100%. Those patients were either very small to create native AVF (*n* = 11) or had already exhausted vascular accesses by thrombosis or central venous occlusion (*n* = 27). Because it is peripheral, the right hepatic vein was the most usual location (close to the puncture site). Due to their critical insertion location, these catheters were replaced monthly on a guidewire and meanwhile displayed several logistic and technical difficulties.

Khallaf et al. conducted a study to emphasize the efficacy of application of TH catheters in 296 HD adult patients [9] while da Motta-Leal-Filho conducted a similar analysis on a small group of 6 patients only [14] and both of the studies reported 100% success rate of TH CVCs. El Gharib et al. conducted a research on 23 patients, one of whom was a child, and reported a considerably lower success rate of 88% [15], the application of TH CVC as a long-term dialysis VA for patients with exhausted classic venous access routes and non-functioning AVF is highly recommended by authors of the 3 studies.

In the present study; CRB (2.58 per 100 catheter days) was the most common complication of TH CVCs. Our incidence of CRB is higher than most other studies conducted on TH CVCs; Khallaf et al. [9] and El Garib et al. [15] reported incidence of 0.15 and 0.13 per 100 catheter days respectively. Furthermore, nearly negligible incidence of CRB (0.22 and 0.18, respectively, per 100 catheter days) was found among the TH CVCs by Stavropoulos et al. [16] and Younes et al. [17].

Optimal exit-site care is essential in order to prevent catheter related infection (CRI) and the choice of the exit-site cleaning agent is critical in pediatric HD patients. Paglialonga et al., [18] observed a significant improvement in CRI after switching from a 5% povidone-iodine solution for exit-site, to a 0.5% chlorhexidine gluconate/70% isopropyl alcohol solution in children undergoing chronic HD through tunneled CVCs.

Antibiotic antimicrobial and combined (antibiotic‐non antibiotic) lock solutions were reported to decrease the incidence of CRI compared to control lock solutions, whereas non‐antibiotic lock solutions reduce CRI only for tunneled CVC [19].

Our increased rate of infection is mostly attributed to the young active age group who are difficult to control their activity together with low hygiene among low socio-economic class of our impoverished patients. In our center; exist site care we use alcohol and betadine and CVC locking by taurolidine and citrate. To the best of our knowledge; TH technique in pediatrics HD patients has not been discussed thoroughly in the literature, nevertheless, by improving catheter care and infection control precautions, we can almost achieve similar outcome to adult studies.

In the present study; 120 tunneled cuffed CVCs were inserted with a mean functioning duration of 215.37 ± 122.7 days and a range of 30—310 days. This functioning duration of our cuffed CVCs was longer than studies, where the average survival time ranged from 116.6 to 157 days [20, 21].

Infection was the most prevalent consequence with cuffed CVCs among our patients, with a frequency of 50%, and incidence of 10.1 episodes per 1,000 catheter days. Cetinkaya et al. [22] reported an increased rate than in many other researches (13.8 percent). On the other hand; Rus et al. [23] reported a very low infection rate (0.9 episodes/1000 catheter days) in astudy done on 31 children in Ljubljana. On comparison of cuffed and uncuffed CVCs, we did not detect significant difference (*p* = 0.209), however, Weijmer et al. showed a low infection incidence (2.9/1,000 catheters/day) among cuffed CVCs and they recommended to use cuffed CVC whenever it can be foreseen that a HD catheter is needed for more than 14 days [24].

MRSA was the most prevalent source of infection in our research, accounting for 66% of cases, with staph aureus accounting for 28%. Many investigations have shown that gram-positive organisms are the most prevalent cause of CRB [13, 20, 25]. Despite the use of lock treatment in our Unit, a high prevalence of infection is observed both cuffed and uncuffed CVCs as discussed above.

AVFs were used to access dialysis in 85 patients. The success rate of AVF among our study group was 97%, which is greater than many other studies. de Souza et al.’ analysis of 10 years of experience in VA for HD children, reported AVF failure rate of 37.8% [26]. Sanabia et al. reported a primary failure rate of 10% in Spain in 1993 [27]. Briones et al. studied the long-term survival of permanent VA in children on HD and found that primary failure occurred in 27% of AVFs and 25% of AVG. The mean duration of AVFs in their study was 2519 months, which was close to the 757.71 ± 512.3 months in the present study [28]. Rus et al. reported a main failure rate of 25.7% [23], which is comparable to rates reported in earlier pediatric studies [29]. Moreover; they found that AVFs were utilized on average 4 months following surgery, which is longer than the time period employed in our research (61.547 ± 10.082 days) [23].

In our analysis, the most common consequence of AVF was thrombosis (13%), whereas 20% of the cases ended well with KT. Similarly; Teixeira et al. reported that 5 out of analyzed 19 AVF had problems in the form of stenosis, aneurysms, or distal ischemia [3]. Other investigations found that problems were more common, such as de Souza et al., who found that thrombosis occurred in 35% of AVFs [26] and Briones et al. who found that thrombosis occurred in 24 out of 79 AVF (30%) [28]. It was suggested in a consensus document by the European Society for Pediatric Nephrology Dialysis Working Group [4] that anti-platelet agents such as aspirin, ticlopidine or clopidogrel, given in the first few months after AVF creation, reduces AVF thrombosis (Grade 2D). They also suggested that tissue plasminogen activator (t-PA) should be used as a catheter locking solution to prevent catheter thrombosis (Grade 2B) and that using t-PA as a thrombolytic agent for CVL thrombosis (Grade 2D).

This study is limited by being single center, and being retrospective in nature with lacking individual details related to different techniques used.

## Conclusion

The development of a native AVF is the preferred VA for pediatric HD patients with the best success rate and the least incidence of complications. When AVF cannot be created or utilized, non-cuffed, citrate-locked CVCs may be suitable as vascular access with change on guide wire until stabilization of long-term VA.Transhepatic CVC can be used as a longterm VA for pediatric patients with exhausted classic venous access routes. Ninety-seven percent (*n* = 182) of the study population had uncuffed temporal catheters inserted as a result of delayed referral. Therefore, it is advised to establish and initiate an AV fistula as soon as possible to prevent vascular access exhaustion from repeated insertion of temporal uncuffed catheters.

For both cuffed and uncuffed CVCs, the most common consequence was catheter-related bacteremia (CRB), (2.58 / 100 catheters day and 10.1 /1000 catheter days respectively) Thus, for pediatric HD patients who must use CVCs as VA for HD, effort should be focused on implementing strict infection control measures.

## Data Availability

The datasets used and/or analysed during the current study are available from the corresponding author on reasonable request.

## Abbreviations

AVF: Arteriovenous fistula
AVG: Arterio-venous grafts
BB: Brachiobaselic
BC: Brachiocephalic
CRB: Catheter-related bacteremia
CUCH: Cairo University Children Hospital
CVC: Central venous catheters
ESKD: End-stage kidney disease
HCV: Hepatitis C virus
HD: Hemodialysis
IQR: Interquartile range
IVC: Inferior vena cava
KT: Kidney transplantation
Lt FV: Left femoral vein
Lt IJV: Left internal jugular vein
MRSA: Methicillin-resistant Staphylococcus aureus
PCR: Polymerase chain reaction
PH1: Primary hyperoxaluria type1
PNU: Pediatric Nephrology Unit
Rt IJV: Right internal jugular vein
Rt IMV: Right internal mammary vein
TH: Trans hepatic
t-PA: Tissue-plasminogen activator
VA: Vascular access
